# Altered monocyte differentiation and macrophage polarization patterns in patients with breast cancer

**DOI:** 10.1186/s12885-018-4284-y

**Published:** 2018-04-03

**Authors:** Chih-Hsing Hung, Fang-Ming Chen, Yi-Ching Lin, Mei-Lan Tsai, Shih-Ling Wang, Yen-Chun Chen, Yi-Ting Chen, Ming-Feng Hou

**Affiliations:** 1 Department of Pediatrics, Kaohsiung Medical University Hospital, Kaohsiung Medical University, Kaohsiung, Taiwan; 2 Department of Pediatrics Faculty of Medicine, College of Medicine, Kaohsiung Medical University, Kaohsiung, Taiwan; 3 Graduate Institute of Medicine, College of Medicine, Kaohsiung Medical University, Kaohsiung, Taiwan; 4 Department of Pediatrics, Kaohsiung Municipal Hsiao-Kang Hospital, Kaohsiung, Taiwan; 5 Research Center for Environmental Medicine, Kaohsiung Medical University, Kaohsiung, Taiwan; 6 Department of Surgery, Kaohsiung Municipal Ta-Tung Hospital, Kaohsiung, Taiwan; 7 Department of Surgery Faculty of Medicine, College of Medicine, Kaohsiung Medical University, Kaohsiung, Taiwan; 8 Division of Breast Surgery, Kaohsiung Medical University Hospital, Kaohsiung, Taiwan; 9 Department of Laboratory Medicine, Kaohsiung Medical University Hospital, Kaohsiung Medical University, Kaohsiung, Taiwan; 10 Department of Laboratory Medicine, Faculty of Medicine, College of Medicine, Kaohsiung Medical University, Kaohsiung, Taiwan; 11 Department of Surgery, Kaohsiung Municipal Hsiao-Kang Hospital, Kaohsiung Medical University, Kaohsiung, Taiwan

**Keywords:** Macrophage, Polarization, M1, M2, Breast cancer, PM-2 K

## Abstract

**Background:**

Macrophage heterogeneity is the main feature of the tumour microenvironment. Breast cancer is one of the most life-threatening cancers. However, macrophage polarization patterns in different tumour stages and the importance of its relationship to human epidermal growth factor receptor 2 (HER2) in breast cancer remains highly unclear. The present study investigated the patterns of monocyte differentiation and macrophage polarization in breast cancer.

**Methods:**

Patients with breast cancer (*n* = 48) and healthy controls (*n* = 39) were prospectively recruited. The percentages and subsets of circulating macrophage-like cells were analysed by flow cytometry, and the polarization patterns of these cells in the peripheral blood of patients with breast cancer were compared with those of healthy controls. In addition, macrophage polarization patterns in different stages and HER2 status in breast cancer were investigated.

**Results:**

The percentages of circulating macrophages, which are defined as PM-2 K^+^ cells in the peripheral blood, were significantly higher in patients with breast cancer than in healthy controls. The percentages of M1-like macrophages were significantly lower, but those of M2-like macrophages were significantly higher in patients with breast cancer than in healthy controls. The percentage of M2c-like macrophages was significantly higher in advanced (stages II and III) breast cancer. However, the patterns of macrophage polarization were not associated with HER2 status in breast cancer.

**Conclusions:**

Aberrant macrophage polarization was observed in breast cancer and was correlated with breast cancer stage. These quantitative data may provide new molecular biomarkers and potential therapeutic targets in breast cancer.

**Electronic supplementary material:**

The online version of this article (10.1186/s12885-018-4284-y) contains supplementary material, which is available to authorized users.

## Background

Macrophages, which can be polarized toward the M1 or M2 phenotype in response to environmental signals, are a key phagocytic cell type and produce factors that connect innate immune responses to the adaptive immune system. Macrophages are dynamic and may first participate in inflammation and then in disease resolution [1, 2]. Under standard culture conditions for human macrophages, PM-2 K is an established marker used to identify mature tissue macrophages and to distinguish macrophages from fibrocytes in monocyte-derived cell populations [3]. In human lung biopsy samples, alveolar macrophages are also PM-2 K^+^ [3].

M1 and M2 macrophages express distinct sets of surface markers and proinflammatory mediators [1, 2, 4, 5]. The phenotypic diversity of macrophages increases with tumour development. Tumour-associated macrophages (TAMs) are an integral component that contributes to tumour growth and progression through many mechanisms in the tumour microenvironment [6]. One of the hallmarks of malignancy is the polarization of TAMs from an M1 proimmune phenotype to an M2 immunosuppressive phenotype. M1–M2 phenotype switching during the early phases of cancer promotes tumorigenesis as well as tumour growth and progression [7]. TAMs in breast cancer are primarily an M2 macrophage subpopulation that promotes tumour progression and metastasis via the release of various cytokines, including chemokines and growth factors [4, 8]. However, the correlation between macrophage polarization patterns and breast cancer stage remains unclear.

In breast cancer, analyses of molecular biomarkers can be valuable to ensure that patients receive current optimal treatment. Established biomarkers, such as the oestrogen receptor, progesterone receptor, human epidermal growth factor receptor 2 (HER2), and Ki67, have been used to classify heterogeneous diseases into several categories to predict prognosis and determine appropriate treatment modalities for individual patients. HER2 is a 185-kDa glycoprotein with tyrosine kinase activity. HER2 protein overexpression or gene amplification occurs in approximately 20%–30% of newly diagnosed advanced breast cancers [9, 10]. HER2 protein overexpression has been considered indicative of an adverse prognosis and is a clinical predictor of treatment response to the humanized monoclonal antibody trastuzumab [11–13].

Although new therapeutic agents have been developed in the past few decades, many patients with breast cancer continue to die due to disease relapse; therefore, novel immunological markers, which can act as specific therapeutic targets, are required. Since macrophages play important roles in tumour immunity, we aimed to evaluate the presence of PM-2 K^+^ cells in the peripheral blood and their relationship with breast cancer, including HER2 status and tumour stage, in the present case–control study using a newly developed multicolour flow cytometric tool. This study led to the novel finding of an altered distribution pattern of circulating PM-2 K^+^ macrophage-like cells, which are associated with advanced breast cancer stages but not with HER2 status in patients with breast cancer.

## Methods

### Study population

Adult patients with breast cancer at the inpatient departments of Kaohsiung Municipal Ta-Tung Hospital and Kaohsiung Medical University Hospital in southern Taiwan were prospectively included in the present study. Patients who satisfied the following inclusion criteria were eligible for prospective enrolment: (1) at least 18 years of age and (2) breast cancer without preoperative systemic therapy. The volunteers who submitted to routine annual physical and laboratory examinations and who did not have a history of cancer were included as healthy controls. The study protocol was approved by the institutional review boards of the study hospitals. After informed consent was provided, peripheral blood samples were obtained from healthy controls and from patients with breast cancer. Case–control comparisons were performed depending on the availability of the samples at the time of analysis.

### Flow cytometric and in vitro analyses of peripheral blood macrophages

A multicolour flow cytometric method was established to identify and distinguish circulating macrophages, which were defined by the expression of PM-2 K in the peripheral blood mononuclear cells (PBMCs) of study patients; doubling cells were eliminated and appropriate fluorescence minus one controls were used. Studies have demonstrated that PM-2 K-stained cells represent mature tissue macrophages and that this marker can be used to distinguish macrophages from fibrocytes in humans [3, 14]. PBMCs were stained with a purified antimacrophage antibody (PM-2 K, AbD Serotec- a Bio-Rad Company, Hercules, CA, USA), followed by antimouse IgG-fluorescein isothiocyanate as previously described [3]. After they were washed, the cells were stained with CD3-Pacific blue (UCHL1, BD Biosciences, San Jose, CA, USA), CD19-Pacific blue (HIB19, eBioscience, San Diego, CA, USA), CD14-PE/Cy7 (61D3, eBioscience), and the appropriate isotype controls [3, 14]. The cells that expressed PM-2 K with or without CD14 expression in non-T and non-B (CD3^−^CD19^−^) cell populations were defined as macrophage-like cells and were divided into PM-2 K^+^CD14^+^ and PM-2 K^+^CD14^−^ subsets for macrophage polarization analysis. The histograms that show the gating strategy for flow cytometry are shown in Additional file 1.

### Definition and phenotypic characterization of circulating macrophage subsets in PBMCs

For the macrophage polarization analysis, a multicolour flow cytometry protocol with a sequential gating strategy was developed as previously described [15]. Cell suspensions were stained with antibodies specific for CCR7 (Miltenyi Biotec, Bergisch Gladbach, Germany), CD86 (BioLegend, Sen Diego, CA, USA), CXCR1 (R&D Systems, Minneapolis, MN, USA), and CCR2 (BioLegend), and the cells were then fixed and permeabilized with Cytofix–Cytoperm solution (BD Biosciences). In the PM-2 K^+^ cell population, cells that were CCR7^+^CD86^+^ were defined as M1-like macrophages, while those that were CCR7^−^CXCR1^+^, CCR7^−^CD86^+^, and CCR7^−^CCR2^+^ were defined as M2a-, M2b-, and M2c-like macrophages, respectively [3, 15]. The histograms with all the antibodies and gating strategies used in flow cytometry for the macrophage subsets are shown in Additional file 1.

### Statistical analysis

Statistical analysis was performed using IBM SPSS Statistics (Version 19, IBM, Armonk, NY, USA) and GraphPad Prism (Version 5, GraphPad Prism Software, Los Angeles, CA, USA). The Mann–Whitney U test was used to determine differences between healthy controls and patients with breast cancer. The Kruskal–Wallis test with a post hoc Dunn multiple comparison test was used to determine differences among the subgroups of patients with breast cancer.

## Results

### The characteristics of patients with breast cancer

In all, 48 patients with breast cancer were enrolled in the present study. The mean age including the standard deviation of patients was 55.54 ± 9.76 years. Among patients, 44 (91.7%) had ductal carcinoma, one (2%) had lobular carcinoma, and 3 (6.3%) had other forms of breast cancer. In terms of cancer stage, 8 (16.7%) patients had stage 0, 24 (50.0%) had stage I, and 16 (33.3%) had stage II or III. Other clinicopathological features are shown in Table 1.

**Table 1:** The clinicopathological features of 48 patients with breast cancer

	Case number	Percent
Age		
< 55-year-old	22	45.8
≥ 55-year-old	26	54.2
Histopathological type		
In situ Ductal carcinoma	6	12.5
In situ Lobular carcinoma	1	2.0
Invasive Ductal Carcinoma	38	79.2
Others*	3	6.3
Stage		
0	8	16.7
I	24	50.0
II	13	27.0
III	3	6.3
IV	0	0.0
Histologic grade		
1	9	18.7
2	26	54.2
3	10	20.8
unclear	3	6.3
Pathological tumor stage		
0#	8	16.6
1	21	43.8
2 & 3	19	39.6
Lymph node status (positive node)		
0	35	72.9
1–3	11	22.9
> 104–9	0	0.0
> 10	2	4.2
Lymph-vascular invasion		
Absent	40	83.3
Present	8	16.7
Human epidermal growth factor receptor 2 (HER2)		
Negative	29	60.4
Positive	19	39.6
Estrogen receptor (ER)		
Negative	11	22.9
Positive	37	77.1
Progesterone receptor (PR)		
Negative	11	22.9
Positive	37	77.1
Ki67		
≦ 20%	20	41.7
> 20%	21	43.8
Non-detection	7	14.5
Received chemotherapy		
No	30	62.5
Yes	18	37.5
Received radiation therapy		
No	16	33.3
Yes	32	66.7
Received target therapy		
No	37	77.1
Yes	11	22.9

*: One is papillary carcinoma, one is mucinous adenocarcinoma and one is Paget disease. #: One of pathological tumor stage 0 is Paget disease

### Increased percentage of PM-2 K^+^ cells in the peripheral blood of patients with breast cancer

**Fig. 1:**
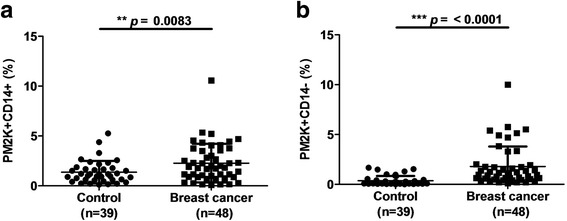
Comparison of peripheral blood macrophages in patients with breast cancer and healthy controls. The percentages of peripheral blood macrophages with (**a**) PM-2 K^+^CD14^+^ expression and (**b**) PM-2 K^+^CD14^−^ expression were significantly higher in patients with breast cancer than in healthy controls. The differences were highly pronounced in the PM-2 K^+^CD14^−^ subset (**b**). **p < 0.01; ***p < 0.001

In the case–control study population, 48 patients with breast cancer and 39 healthy controls were enrolled in the present study. No significant differences were observed in age distribution. The percentages of macrophage-like cells in the peripheral blood, either in the PM-2 K^+^CD14^+^ (*p* = 0.0083; Fig. 1a) or the PM-2 K^+^CD14^−^ subset (*p* < 0.0001; Fig. 1b), were significantly higher in patients with breast cancer than in healthy controls. Pronounced differences were observed in the PM-2 K^+^CD14^−^ subset between breast cancer patients and controls.

### Altered patterns of macrophage polarization in patients with breast cancer

**Fig. 2:**
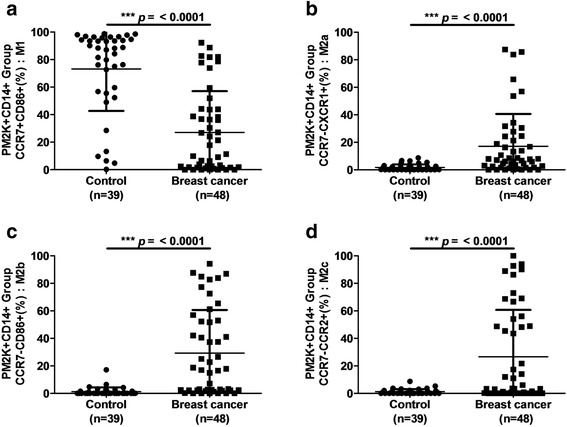
Comparison of M1-, M2a-, M2b-, and M2c-like macrophages in peripheral blood PM-2 K^+^CD14^+^ cells in patients with breast cancer and healthy controls. (**a)** The percentages of M1-like macrophages in the population of PM-2 K^+^CD14^+^ cells were significantly lower in patients with breast cancer than in healthy controls. The percentages of (**b**) M2a-, (**c**) M2b-, and (**d**) M2c-like macrophages in the population of PM-2 K^+^CD14^+^ cells were significantly higher in patients with breast cancer than in healthy controls. ***p < 0.001

**Fig. 3:**
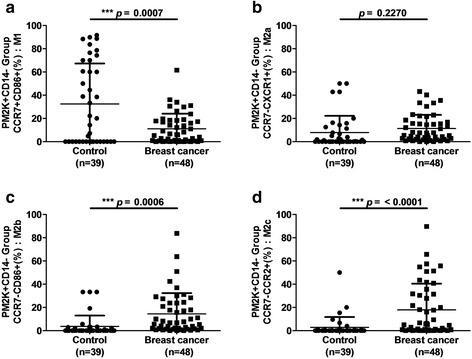
Comparison of M1-, M2a-, M2b-, and M2c-like macrophages in peripheral blood PM-2 K^+^CD14^−^ cells in patients with breast cancer and healthy controls. (**a**) The percentages of M1-like macrophages out of the PM-2 K^+^CD14^−^ cells were significantly lower in patients with breast cancer than the in healthy controls. (**b**) The percentages of M2a-like macrophages out of the PM-2 K^+^CD14^−^ cells were not significantly different between patients with breast cancer and healthy controls. The percentages of (**c**) M2b- and (**d**) M2c-like macrophages out of the PM-2 K^+^CD14^−^ cells were significantly higher in patients with breast cancer than in healthy controls. ***p < 0.001

In the PM-2 K^+^CD14^+^ cell population, the percentage of M1-like macrophages that were CCR7^+^CD86^+^ was significantly lower in patients with breast cancer than in healthy controls (*p* < 0.0001; Fig. 2a). However, the percentages of M2a- (p < 0.0001; Fig. 2b), M2b- (p < 0.0001; Fig. 2c), and M2c-like macrophages (p < 0.0001; Fig. 2d) in the PM-2 K^+^CD14^+^ cell population were significantly lower in patients with breast cancer than in healthy controls. In the PM-2 K^+^CD14^−^ subset, the percentage of M1-like macrophages was significantly lower in patients with breast cancer than in healthy controls (*p* = 0.0007; Fig. 3a). The percentages of M2b- (*p* = 0.0006; Fig. 3c) and M2c-like macrophages (p < 0.0001; Fig. 3d) but not those of the M2a-like macrophages (*p* = 0.2270; Fig. 3b) of the PM-2 K^+^CD14^−^ cell population were significantly higher in patients with breast cancer than in healthy controls. These results suggested that patients with breast cancer exhibit a distinct pattern of macrophage polarization in the peripheral blood, namely a lower percentage of M1-like macrophages but a higher percentage of M2-like macrophages, than those of healthy controls. The percentages of circulating macrophage subsets in patients with breast cancer and in healthy controls are listed in Table 2.

**Table 2:** The percentages of circulating macrophage subsets in patients with breast cancer and the healthy controls

Subsets	PM2 K^+^ cells		PM2 K^+^CD14^+^ cells				PM2 K^+^CD14^−^ cells			
	PM2 K^+^CD14^+^	PM2 K^+^CD14^−^	M1	M2a	M2b	M2c	M1	M2a	M2b	M2c
Control (%, mean ± SD)	1.37 ± 1.26	0.37 ± 0.47	73.24 ± 30.54	1.72 ± 2.23	1.25 ± 3.02	1.23 ± 1.80	32.46 ± 34.81	7.92 ± 14.32	3.66 ± 9.27	2.86 ± 8.85
Breast Cancer (%, mean ± SD)	2.27 ± 1.93*	1.79 ± 2.00*	27.15 ± 29.93*	17.12 ± 23.49*	29.30 ± 31.30*	26.63 ± 34.08*	11.01 ± 13.08*	11.31 ± 11.70	14.36 ± 17.98*	17.88 ± 22.60*
Aberrance in breast cancer	**↑**	**↑**	**↓**	**↑**	**↑**	**↑**	**↓**	n.s	**↑**	**↑**

*: *p* < 0.05

n.s: not significant

### Altered patterns of macrophage polarization are associated with breast cancer stage

**Fig. 4:**
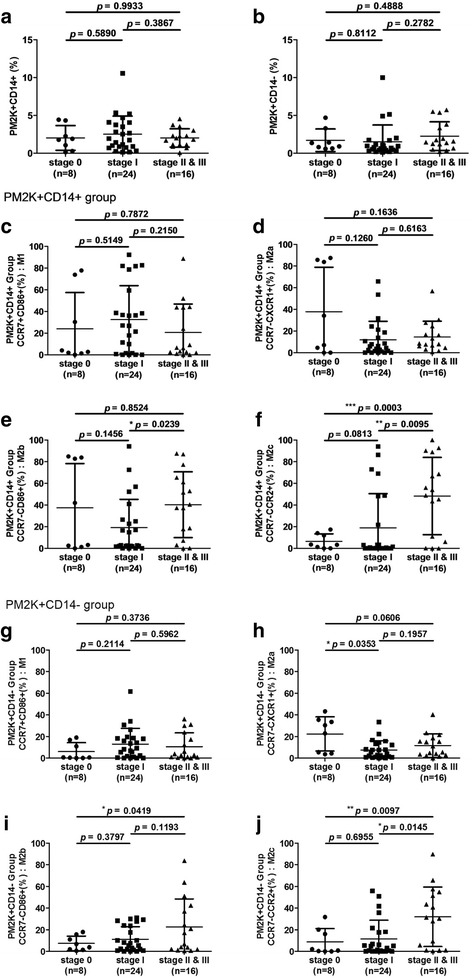
Relationship between macrophage polarization patterns and breast cancer stages. The percentages of peripheral blood macrophages with neither (**a**) PM-2 K^+^CD14^+^ expression nor (**b**) PM-2 K^+^CD14^−^ expression were significantly different between patients with advanced breast cancer (stages II and III) and those with early (stages 0 and I) breast cancer. In PM-2 K^+^CD14^+^ cells, the percentages of (**c**) M1-, (**d**) M2a and (**e**) M2b-like macrophages in patients with different breast cancer stages were not significantly different. (**f**) The percentages of M2c-like macrophages in the PM-2 K^+^CD14^+^ subset were significantly higher in patients with advanced breast cancer than in those with early breast cancer. In PM-2 K^+^CD14^−^ cells, the percentages of (**g**) M1-like macrophages did not differ significantly according to breast cancer stage. The percentage of (**h**) M2a-like macrophages in the PM-2 K + CD14- subset was higher in patients with stage 0 than in those with stage I, and (**i**) M2b-like macrophages was lower in patients with stage 0 than in those with advanced breast cancer (stage II and III). (**j**) The percentage of M2c-like macrophages in the PM-2 K^+^CD14^−^ subset was significantly higher in patients with advanced breast cancer (stages II and III) than in those with early breast cancer (stages 0 and I). *p < 0.05; **p < 0.01; ***p < 0.001

We evaluated the patterns of macrophage polarization in patients with different stages of breast cancer. In the PM-2 K^+^CD14^+^ and PM-2 K^+^CD14^−^ subsets, the percentages of macrophage-like cells in the peripheral blood did not differ significantly between patients with advanced (stages II and III) breast cancer and those with early (stages 0 and I) breast cancer (Fig. 4a and b). In the PM-2 K^+^CD14^+^ subset, the percentages of M1- and M2a-like macrophages did not differ significantly between early and advanced breast cancer (Fig. 4c and d). The percentage of M2b-like macrophahes was lower in stage I (Fig. 4e), and the percentage of M2c-like macrophages was significantly higher in advanced (stages II and III) breast cancer (Fig. 4f). We next examined the PM-2 K^+^CD14^−^ subset. In the PM-2 K^+^CD14^−^ cell population, the percentage of M1- like macrophages was not significantly different between patients with early and advanced breast cancers (Fig. 4g). The percentage of M2a-like macrophages was higher in stage 0 (Fig. 4h), but the percentages of M2b- and M2c-like macrophages were higher in stages II and stage III breast cancer (Fig. 4i and j).

### Correlation between macrophage polarization patterns and HER2 status in breast cancer

Studies have suggested that serum HER-2 is a candidate marker for breast cancer [9, 10]; therefore, we investigated the correlation between the patterns of macrophage polarization and serum HER-2 status in breast cancer. The percentages of macrophage-like cells did not differ significantly between patients with HER2-postive and those with HER2-negative breast cancer. In the PM-2 K^+^CD14^+^ and PM-2 K^+^CD14^−^ subsets of macrophage-like cells, the percentages of M1-, M2a-, M2b-, and M2c-like macrophages were not significantly different between patients with HER2-positive and those with HER2-negative breast cancer (Additional file 2).

## Discussion

Macrophages are critical immune cells and important regulators of inflammatory processes. Resident macrophages can act as sensors for tissue damage and can maintain tissue homeostasis. Although molecular markers, such as the oestrogen receptor, progesterone receptor, HER2, Ki67, and DNA ploidy, have been used to classify heterogeneous diseases into several categories to predict prognosis and determine treatment modalities [16, 17], current diagnoses and therapies are inadequate because numerous patients die due to disease relapse. Therefore, to improve disease diagnosis, novel molecular markers are required for therapeutic strategies, gene expression, and microRNA profiling [18, 19]. Several studies have identified novel biomarkers among cell cycle regulators, oncogenes, and tumour suppressor genes that are critically involved in carcinogenesis in an attempt to improve breast cancer diagnosis and treatment. Here, we describe an altered pattern of circulating monocyte differentiation and macrophage polarization in patients with breast cancer. Moreover, the molecular nature of the PM-2 K marker has allowed its use in the distinction of macrophage populations [3]. The present results showed that the percentages of macrophages, in both the PM-2 K^+^CD14^+^ and PM-2 K^+^CD14^−^ subsets, were significantly higher in patients with breast cancer than in healthy controls. However, the percentages of M1 macrophages in the peripheral blood were significantly lower in patients with breast cancer than in healthy controls, which might indicate poor antitumor activities of circulating macrophages in patients with breast cancer. In addition, the percentages of M2 macrophages were significantly higher in patients with breast cancer than in healthy controls. This suggests that patients with breast cancer may have a greater number of macrophages with M2 phenotypes, such as TAMs, which promote breast cancer progression and metastasis via the release of various cytokines, including chemokines and growth factors.

Macrophage polarization with cytokine release connects innate immune responses to the adaptive immune system. Polarized macrophages are broadly classified as M1 or M2 macrophages. Interleukin (IL)-4 polarization, referred to as either alternative or M2a activation, promotes a response characteristic of wound healing and parasite immunity, whereas interferon-r polarization, known as classical or M1 activation, programs monocytes for intracellular killing and tumour resistance [20, 21]. M2 macrophages are further divided into three subsets: M2a, induced by IL-4 or IL-13; M2b, induced by immune complexes and agonists of toll-like receptors or IL-1 receptors; and M2c, induced by IL-10 and glucocorticoid hormones [15, 20]. IL-10-producing cells play a crucial role in tumour development [22]. A novel and important finding in the present study is the correlation between macrophage polarization patterns and breast cancer stage. A significantly higher percentage of the M2c subset was observed in patients with advanced (stages II and III) breast cancer than in those with early (stages 0 and I) breast cancer. A higher percentage of M2c cells, which produce higher levels of IL-10, may induce progression to advanced breast cancer stages. These findings suggest that a higher percentage of cells with the M2 anti-inflammatory phenotype in patients with advanced breast cancer may promote tumorigenesis and tumour progression [4, 8].

HER2 is expressed in most in situ breast cancers but is maintained in only 20%–30% of invasive breast cancers. During breast cancer tumorigenesis, a progressive loss of HER2 expression from benign to ductal carcinoma in situ was observed, with an almost complete loss of HER2 expression in invasive breast cancer. Thus, the exact role of HER2 in breast cancer has yet to be completely defined [23]. The results of the present study demonstrated that the percentages of macrophages in both the PM-2 K^+^CD14^+^ and PM-2 K^+^CD14^−^ subsets were not different between patients with HER2-positive breast cancer and those with HER2-negative breast cancer. When the subsets of macrophages were stratified by M1 and M2 markers, no significant differences were observed in the patterns of macrophage polarization between patients with HER2-positive breast cancer and those with HER2-negative breast cancer.

Currently, there is still a lack of clinically efficacious biomarkers of breast cancer that can be used to monitor disease activity, progression, severity, and therapeutic outcomes. Although HER2 status is used as a marker, it is a relatively nonspecific response marker and cannot be used to define highly specific disease phenotypes and disease severity. Therefore, clinically efficacious markers with easy accessibility are required to facilitate the diagnosis of advanced breast cancer and to predict its severity and control status. Our study, which had a relatively small sample size, supports the use of multicolour flow cytometry to identify advanced breast cancers from a small amount of peripheral blood via the detection of circulating macrophages and their polarization patterns on the basis of PM-2 K and polarization markers. In the present study, higher percentages of total and M2 macrophages and lower percentages of M1 macrophages were found in patients with breast cancer. Moreover, the percentage of cells in the M2c subset was higher in patients with advanced stages (stage II and III) of breast cancer than in patients with early stages (stage 0 and I) of breast cancer. Aberrant macrophage polarization might facilitate the development of new biomarkers, and M2c macrophages might be a potential therapeutic target in advanced breast cancer. However, research on the correlations among the different subtypes of macrophages, tissue and circulating cells, as well as functional studies on the effects of different drugs on macrophage polarization in breast cancer might be required in the future.

## Conclusions

In the present study, we provide a new approach for breast cancer diagnosis and a potentially novel target for the investigation of the pathogenic mechanisms of advanced breast cancer. Aberrant macrophage polarization was found in breast cancer and was correlated with the stages but not with HER2 status. These quantitative data may facilitate the evaluation of the clinical utility of macrophage polarization patterns in a larger study population of patients with breast cancer. Furthermore, this analytical platform may be generally applicable to various diseases if macrophage polarization patterns are known to be important in the pathogenesis of the targeted diseases.

## Additional files

Additional file 1: The gating strategy and histograms with the gating of all the antibodies by flow cytometry. Sample data from a patient with breast cancer are shown. Live cells were gated on a forward scatter (FSC)/side scatter (SSC) plot (A). These cells were then further gated to determine CD3^−^, CD19^−^ (B), PM-2 K^+^ macrophages (C). Macrophages were further gated to determine CCR7^+^CD86^+^ M1-like macrophages, CCR7^−^CXCR1^+^ M2a-like macrophages, CCR7^−^CD86^+^ M2b-like macrophages, and CCR7^−^CCR2^+^ M2c-like macrophages in PM-2 K^+^CD14^+^ (D) and PM-2 K^+^CD14^−^ (E) groups. (PDF 46 kb)

Additional file 2: Relationship between macrophage polarization patterns and HER2 status in breast cancer. The percentages of peripheral blood macrophages with neither (a) the PM-2 K^+^CD14^+^ expression profile nor (b) the PM-2 K^+^CD14^−^ expression profile were significantly different between patients with HER2-positive breast cancer and those with HER2-negative breast cancer. In the PM-2 K^+^CD14^+^ and PM-2 K^+^CD14^−^ subsets, the percentages of M1-, M2a-, M2b-, and M2c-like macrophages were not significantly different between patients with HER2-positive breast cancer and those with HER2-negative breast cancer (c–j). (PDF 108 kb)

## Abbreviations

HER2: human epidermal growth factor receptor 2
IL: interleukin
PBMCs: peripheral blood mononuclear cells
TAM: tumour-associated macrophage
