# Supplementary figures and images for: Altered monocyte differentiation and macrophage polarization patterns in patients with breast cancer
Source: BMC Cancer. 2018 Apr 3;18:366. doi: 10.1186/s12885-018-4284-y (PMC5883269; doi:10.1186/s12885-018-4284-y)

(A)

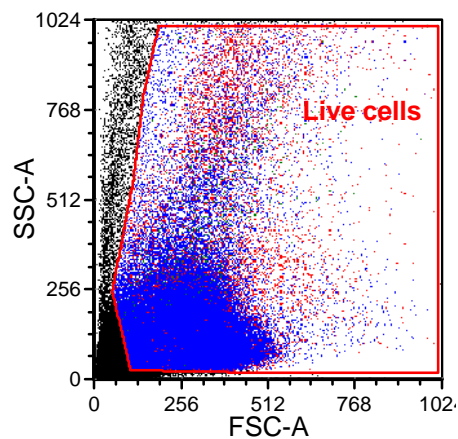

(B)

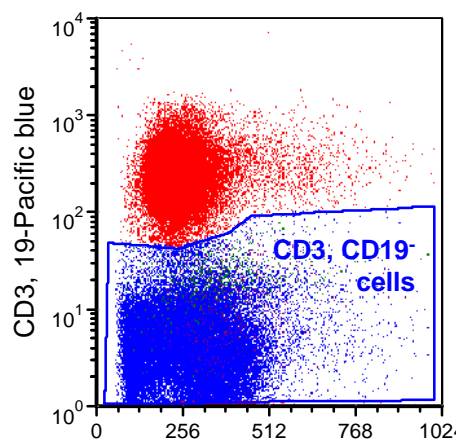

(C)

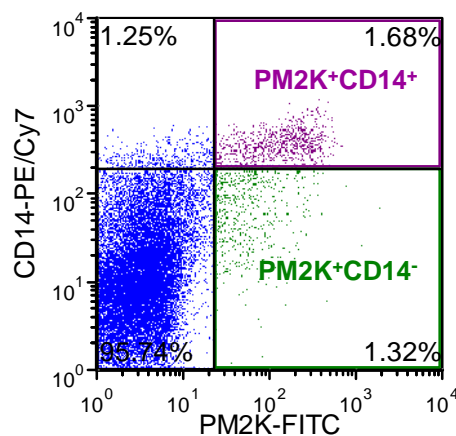

(D)

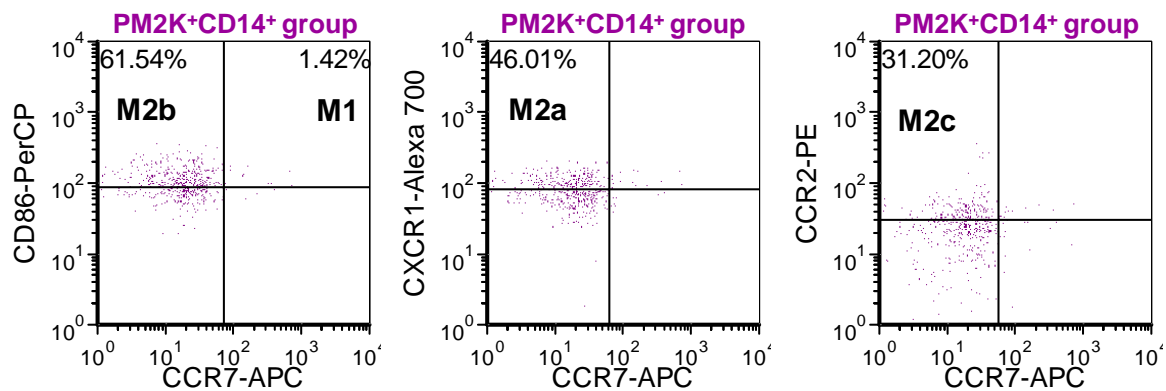

(E)

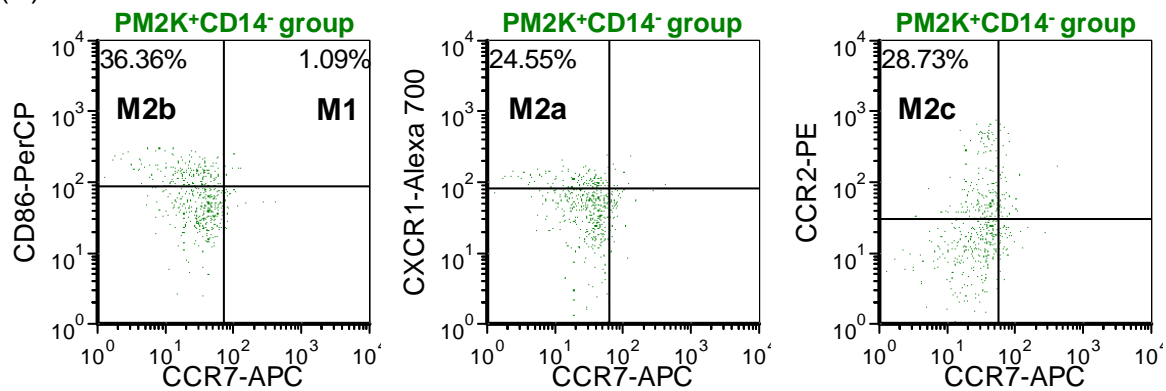

Supplement: Supplementary file 1 — The gating strategy and histograms with the gating of all the antibodies by flow cytometry. Sample data from a patient with breast cancer are shown. Live cells were gated on a forward scatter (FSC)/side scatter (SSC) plot (A). These cells were then further gated to determine CD3−, CD19− (B), PM-2 K+ macrophages (C). Macrophages were further gated to determine CCR7+CD86+ M1-like macrophages, CCR7−CXCR1+ M2a-like macrophages, CCR7−CD86+ M2b-like macrophages, and CCR7−CCR2+ M2c-like macrophages in PM-2 K+CD14+ (D) and PM-2 K+CD14− (E) groups. (PDF 46 kb) [file 12885_2018_4284_MOESM1_ESM.pdf]

(a)

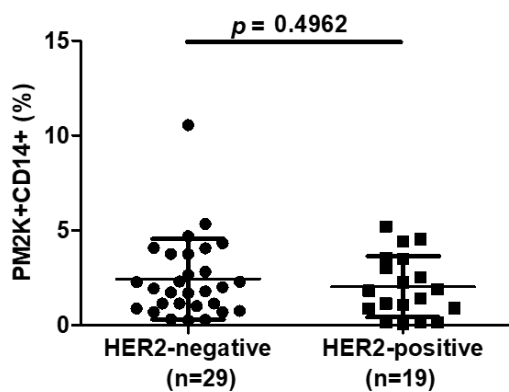

PM2K+CD14+ group

(b)

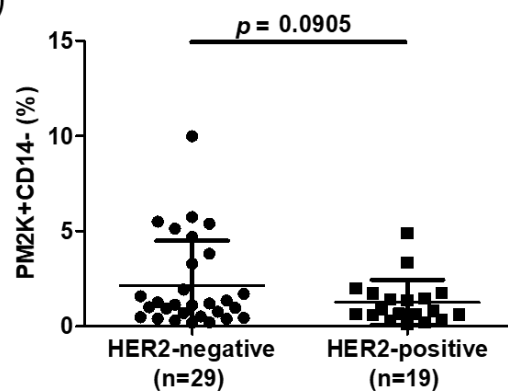

(c)

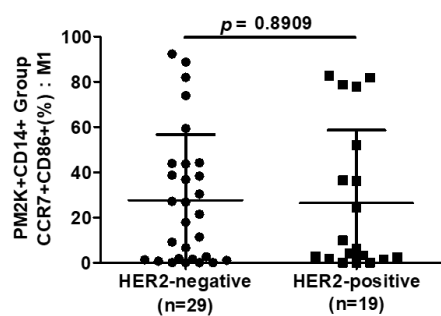

(d)

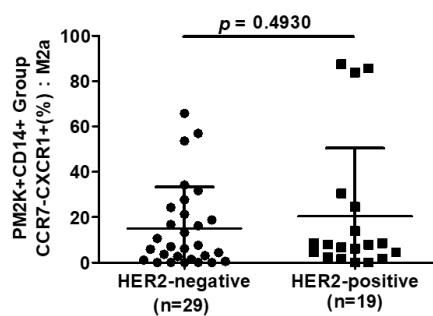

(e)

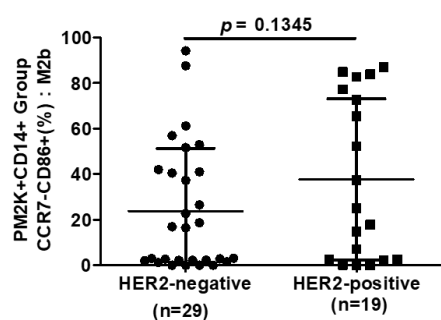

(f)

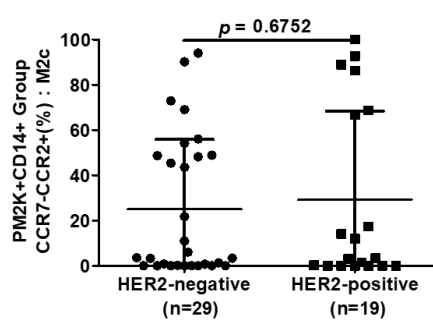

PM2K+CD14- group

(g)

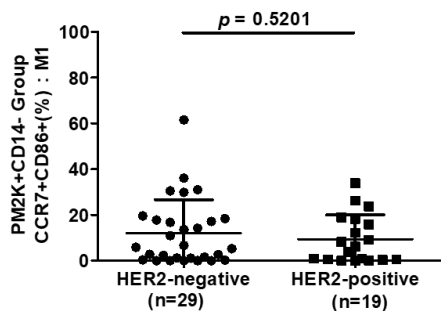

(h)

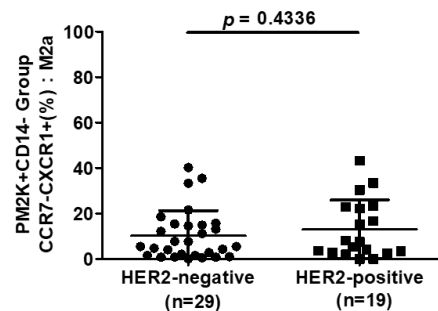

(i)

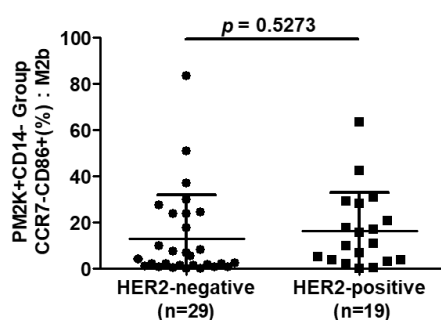

(j)

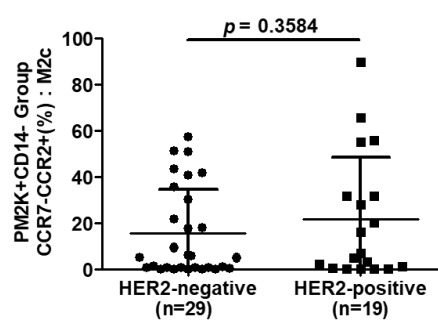

Supplement: Supplementary file 2 — Relationship between macrophage polarization patterns and HER2 status in breast cancer. The percentages of peripheral blood macrophages with neither (a) the PM-2 K+CD14+ expression profile nor (b) the PM-2 K+CD14− expression profile were significantly different between patients with HER2-positive breast cancer and those with HER2-negative breast cancer. In the PM-2 K+CD14+ and PM-2 K+CD14− subsets, the percentages of M1-, M2a-, M2b-, and M2c-like macrophages were not significantly different between patients with HER2-positive breast cancer and those with HER2-negative breast cancer (c–j). (PDF 108 kb) [file 12885_2018_4284_MOESM2_ESM.pdf]
